# Low-cost system for sunlight incidence angle measurement using optical fiber

**DOI:** 10.1016/j.ohx.2022.e00302

**Published:** 2022-03-30

**Authors:** J.S. Botero-Valencia, E. Ospina-Rojas, M. Mejia-Herrera, D. Gonzalez-Montoya, M. Durango-Flórez, C.A. Ramos-Paja

**Affiliations:** a Grupo de Sistemas de Control y Robótica, Instituto Tecnológico Metropolitano, Medellín, Colombia; b Facultad de Minas, Universidad Nacional de Colombia, Medellín, Colombia

**Keywords:** Digital signal processing, Irradiance incident angle, Optical fiber, Photovoltaic systems, Sunlight, Renewable energy

## Abstract

The development and optimization of renewable energy systems are some of the most necessary topics to advance towards secure and sustainable energy models. Photovoltaic energy is one of those sustainable options that could contribute to the reduction of greenhouse gas emissions. The optimal angle of solar incidence producing the highest absorption in a day is an important parameter to install photovoltaic systems. This value is often estimated using simulation models based on geographic location; however, those models ignore the influence of nearby obstruction objects, albedo, and local weather conditions. Such a problem is addressed in this work by designing a system to estimate the optimum angle of solar incidence for the photovoltaic panels. The system is based on an arrangement of 33 measurement points spaced in arcs every 45 degrees in azimuth and every 22.5 degrees in elevation, which provides a wide range for analysis. The light captured by each optical fiber is transmitted to a flat array where the power is measured using a single RGB camera.

## Graphical abstract

**Figure:**
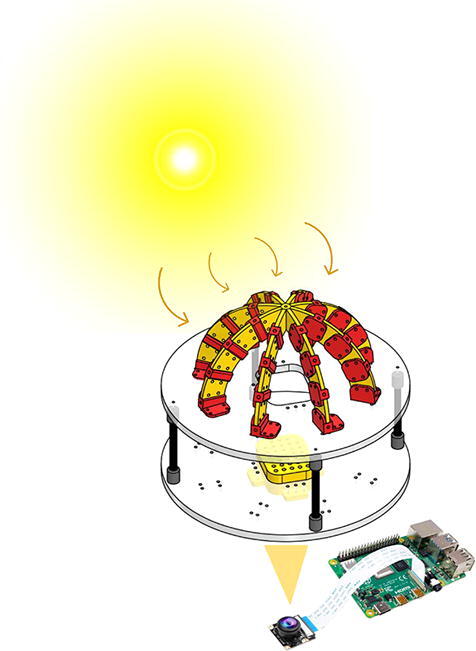

**Specifications table:**

**Table:** 

**Hardware name**	Low-cost system for sunlight incidence angle measurement
**Subject area**	•Engineering
	•Instrumentation
	•Internet of things
**Hardware type**	•Measuring physical properties and in-lab sensors
	•Field measurements and sensors
	•Electrical engineering and computer science
**Open source license**	Creative Commons Attribution-ShareAlike license
**Cost of hardware**	USD 203.91
**Source file repository**	https://doi.org/10.17605/OSF.IO/54KWN

## 1 Hardware in context

The increment of the energy demand has been supplied by different energy sources, where the largest part is covered by nuclear energy and fossil fuels like oil, which creates health problems and contribute to the unsuitability of the planet’s climate [1]. For this reason, renewable energy sources such as wind, biomass, and solar are promising alternatives due to their sustainable, safe, and environmentally friendly operation [2]. In particular, photovoltaic (PV) energy is one of the most implemented types of renewable sources [3]. In these systems, the power delivered depends on environmental variables such as solar irradiance, temperature, and position of the PV panel (i.e panel orientation, inclination, zone of installation, among others) [4], [5], [6]. Different works have analyzed the effect of the tilt angle on the delivered PV power, demonstrating that an optimized tilt angle increases the maximum energy extraction [7], [8]. For example, the work reported in [9] shows that the optimal tilt angle depends on the season and the latitude of the PV installation. Therefore, different models have been developed to calculate the appropriate tilt angle based on the solar irradiance, ground reflectance, and the inclination with the surface.

Another factor related to the energy yield is the partial shading phenomenon, which is caused by trees, birds, or surrounding buildings. Such a phenomenon produces variable energy drops since shaded (or partially shaded) PV cells operate at the second quadrant, thus absorbing energy instead of producing it [10], [11]. Several mechanisms have been proposed to overcome this drawback such as solar collectors or sun trackers [12], [13], which are alternatives to mitigate the power reduction at sunset and nightfall by capturing as much sunlight as possible. In this way, the authors of [14] developed a mathematical model to predict the optical performance of an asymmetric stationary compound parabolic concentrator (ACPC), which was used to adjust an ACPC to increase the electrical production by 2% due to the reduction of the PV cells temperature, thus validating the model. Another approach was presented in [15], which reports the developed of a low-cost sun tracking system based on a LM324 operational amplifier, the moving base of a satellite receiver antenna, and a H-type drive as a base. That solution achieved an increment in the output power of 57% in comparison with a fixed PV panel, where the simple mechatronics device introduced an additional cost of about 15%, thus demonstrating both the low-cost nature of the device and the efficiency improvement for the PV array. Other types of solar collectors are described in [16], which proposes a model to obtain the electrical characteristics of a PV array under partial shading conditions and different orientations, thus taking into account the reverse voltage effect in the power. In particular, for the different panel orientation case, the authors modeled the PV modules using two irradiance variables, thus producing a two-variable analytical model for each PV panel.

Another option to properly calculate the tilt angle and orientation of a PV system are commercial devices with light sensors. Those devices are commonly used in climate stations and for the power estimation of PV installations, but those devices could also be used to estimate the effect of the tilt angle into the power production on a particular zone. Instruments such as the LUXSOLAR L864-LXS-SOL Medium Intensity Obstruction Light [17] and the SunEye 210 are measurement tools for solar site assessment and. In the case of the SunEye, it can measure the available solar energy by day, month, and year intervals, also determining the shading patterns of the installation site [18]. Another interesting tool is the SunEarth [19], which enables to obtain the trajectory and position of the sun through the year for a particular geographic location. It is also very important to design easily accessible tools to support the development of renewable energies as a more open field, which also contributes to fulfill the Sustainable Development Goal 7 (SDG7 - Affordable and clean energy). This is a very important Goal since the generation of clean energy is fundamental for human society and environment conservation.

Despite the previous devices are easy to use, those devices might be expensive, which could increase the total cost of a PV installation. Instead, devices like the one proposed in [20] provide a satisfactory trade-off between accuracy and cost. Such a device can be also used to analyze partial shading conditions and tilt angle effect, thus providing a low-cost alternative for the pre-design stage of a PV system. The device reported in this work improves the work presented in [20] by preserving the low-cost condition, but increasing the number of available tilt sensors to improve the resolution (from 9 to 33 measures). However, the operation principle of this new device is different, since fiber optics are adopted to capture the light instead of individual sensors. Finally, the information from the 33 measurements is used to calculating the optimum tilt angle and the solar irradiance distribution. Moreover, the those measurements can also be used to detect the shading profiles and its possible effects on the PV panels. Therefore, this device provides valuable information needed in the pre-design stage of a PV installation.

## 2 Hardware description

**Fig. 1:**
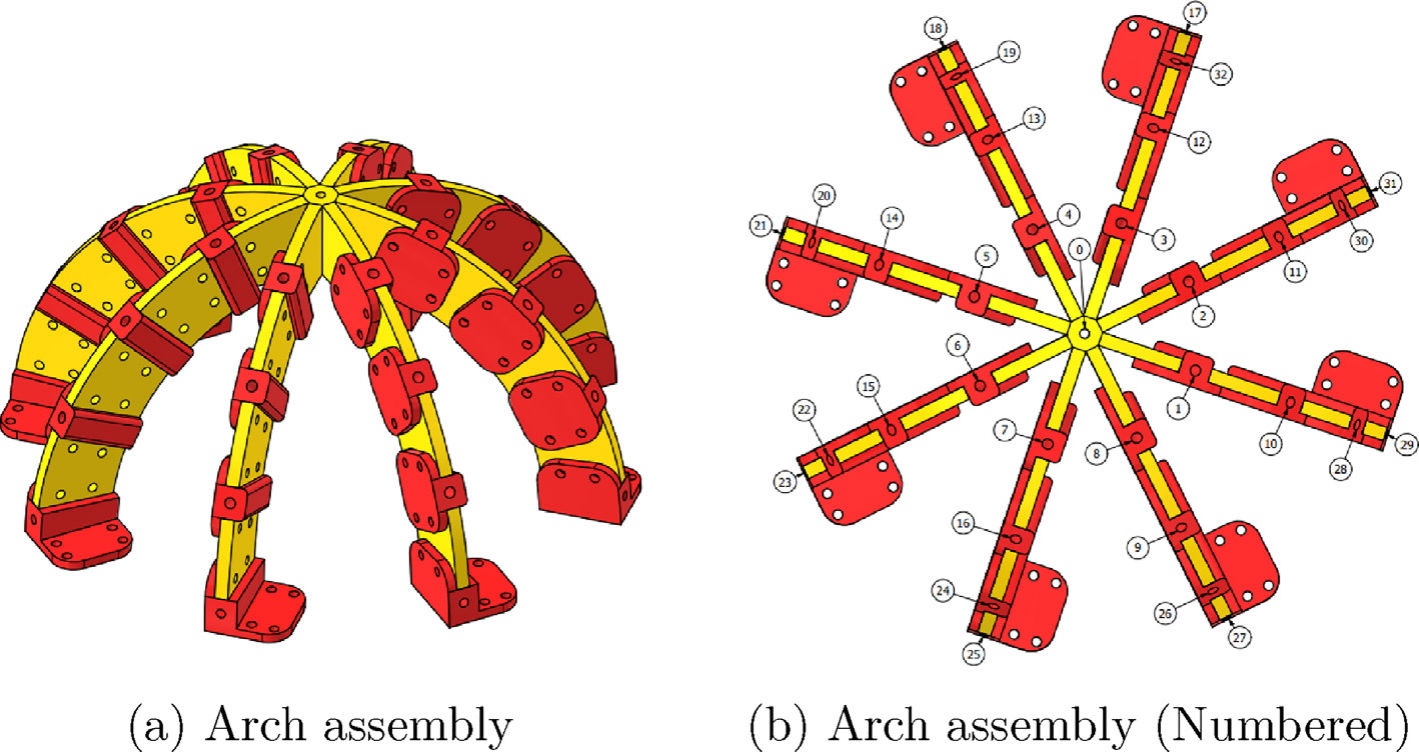
CAD of the assembled system.

The system for sunlight incidence angle measurement is based on an optical fiber array. These fibers are achromatic, which means that although they have an attenuation of the light entering them, it is the same for the wavelengths in the visible spectrum. In general, those optical fibers are composed of glass or plastic filaments, and are commonly used for information transmission based on light beams traveling between their ends, even if the filament is bent. Taking advantage of the optical fiber capacity to capture and transmit light, an array of 33 optical fibers was used on this device. The fibers were distributed to form arcs every 22.5°in order to detect the light intensities at different angles. The mechanical structure that supports the optical fiber comprises four arcs of 23 cm in diameter, which were printed in Polylactic Acid (PLA). The whole structure is assembled with M3 screws, and each of the fibers is numbered to be referenced in an image detection step. Fig. 1a and b show the final assembly and a bottom view, where the numbering that relates the fibers with the position in the matrix shown in the Fig. 3, is presented.

A transparent dome is used to protect the system. Such a dome is made of a thermoformed acrylic that is 3 mm thick and 25 cm in diameter. Moreover, the material was selected to avoid interference with the light measurements, thus its transmittance characteristics do not affect the passage of light. An acrylic base is perforated to serve as a base for the arches, in which the matrix collecting the fibers is located (Fig. 3). The optical fiber used in this system has a PVC coating to protect it from the environment and prevent light from entering from other directions.

**Fig. 2:**
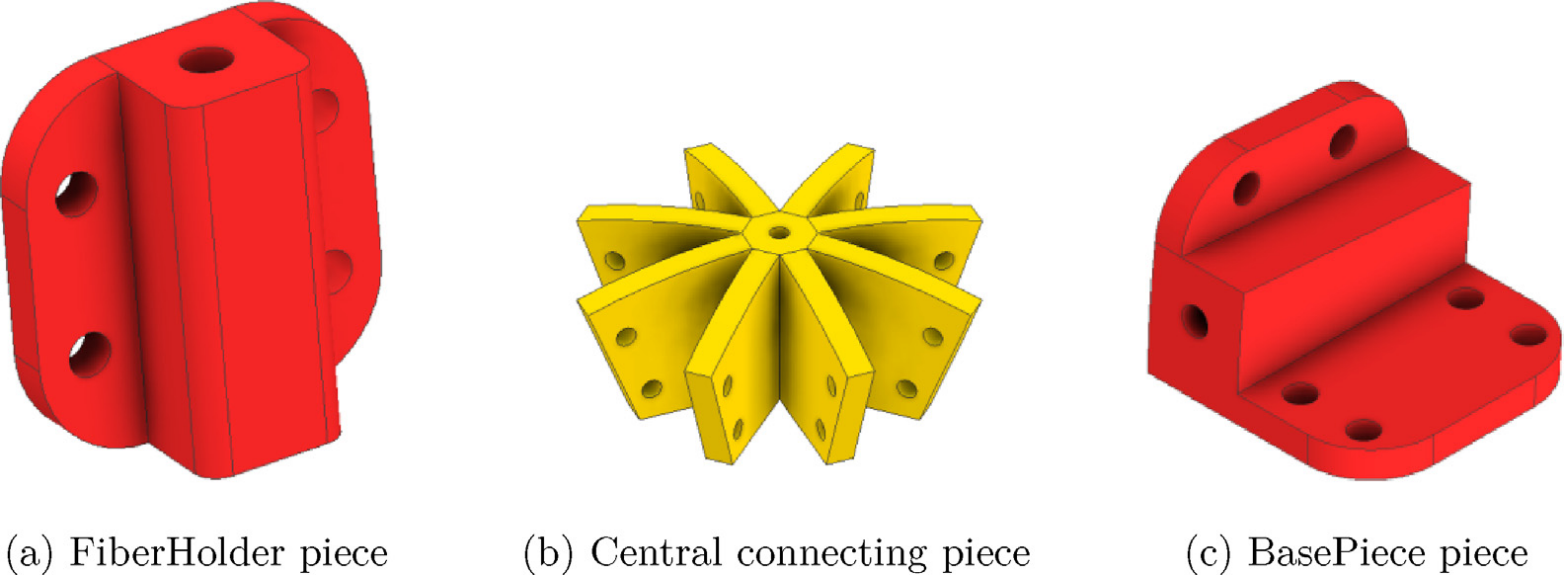
Arch assembly parts.

The arcs were assembled with three different pieces, which are named FiberHolder, BasePiece, and UnionPiece. The optical fiber is inserted into the FiberHolder pieces, to keep the fibers aligned, the BasePiece piece is used to fix the optical fiber at the end of the arches and to fix the structure; those are depicted in Fig. 2a and c. To keep the fibers at 22.5°and shape the arcs, a connecting piece called UnionPiece was used. The part that joins all the arcs and supports the fiber that references 90°is called CenterPiece, and it has the function of joining the eight semi-arcs to form four arches in total. Such a CenterPiece is depicted in Fig. 2b.

**Fig. 3:**
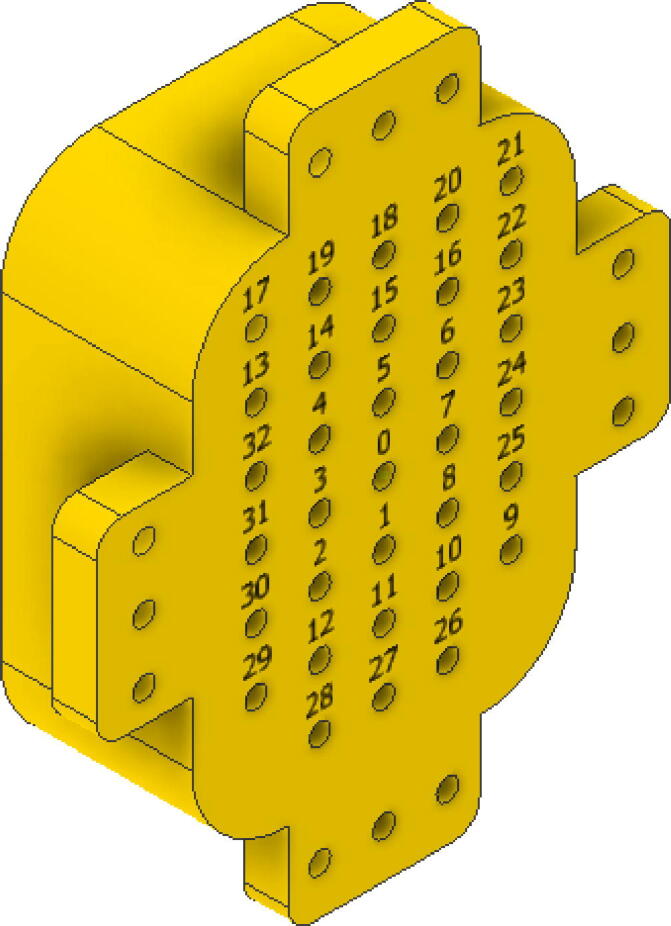
Fiber optic holder matrix.

The optical fiber parts have 23 cm long, where the end that is not attached to the arches is inserted into a matrix also printed in PLA; such a matrix part is depicted in Fig. 3. Below this matrix, a camera is located to capture an image of the matrix with the fibers. Depending on the points that show higher light intensity, the angle of sunlight incidence can differ, for this reason, the matrix points were numbered. Finally, the data captured by the camera is processed on a Raspberry Pi3, which can be replaced by other computer system. As the system will be exposed to the environment, the acrylic hemisphere is used to cover the entire structure to protect the device.

## 3 Design files

**Fig. 4:**
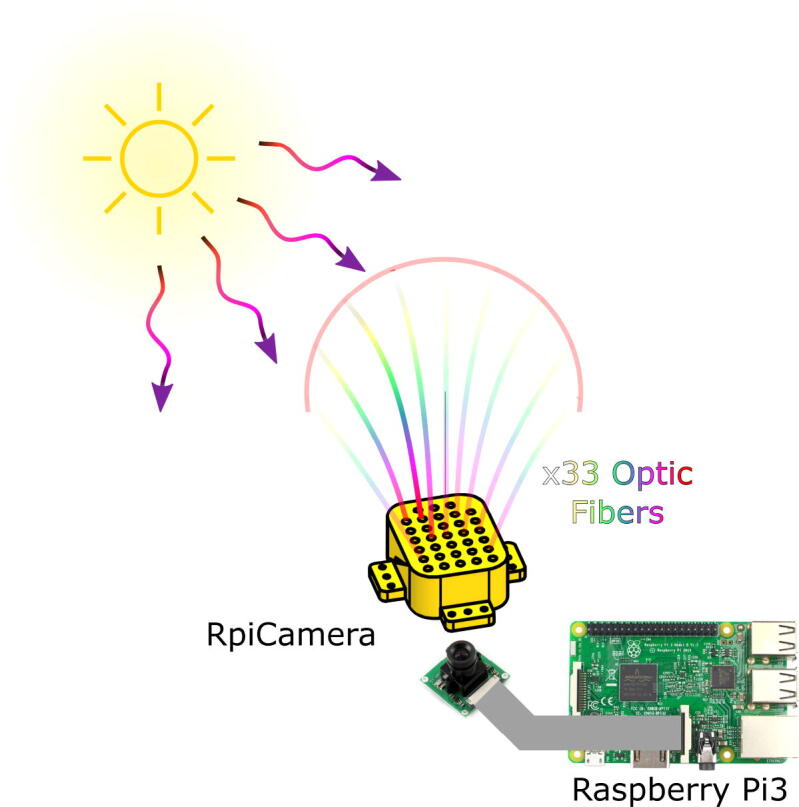
Graphic representation of the overall operation of the proposed system.

This section presents each of the parts designed with a Computer-Aided Design (CAD) software. Each part is available in the repository in STEP format, thus it can be printed using fused deposition material (FDM) or any other 3D printing technology. In this case, the parts were 3D printed in PLA with a hexagonal infill of 35%. Each piece performs a specific function and should be used as explained below to reproduce the system proposed in this article. However, since the digital version of the files is available, it is also possible to make modifications oriented to add additional hardware. Fig. 4 presents a graphical representation of the system operation, which uses 33 optical fibers in specific angles to analyze the solar incidence on the system. The system uses a hemispherical arrangement that positions each optical fiber to examine the solar incidence through a camera. In this case, a Raspberry Pi camera with a Flexible Flat Cable (FFC) connection was used. However, the system can be constructed with any other independent camera or mobile device.

## 4 Design files summary

The system consists of eight arms, each arm positions four fibers, using three *UnionPiece*, three *FiberHolder*, and one *BasePiece*. This section describes each of the files necessary for the construction of this device. The Table 1 reports the file name, license and file location; all files are available in STEP format.

**Table 1:** Design files

Design file name	Open source license	Location of the file
CenterPiece.step	GNU GPL v3.	https://osf.io/8bqs3/
UnionPiece.step	GNU GPL v3.	https://osf.io/y93jx/
FiberHolder.step	GNU GPL v3.	https://osf.io/dmpqw/
BasePiece.step	GNU GPL v3.	https://osf.io/avb6p/
Base.step	GNU GPL v3.	https://osf.io/d3buk/
FiberMatrix.step	GNU GPL v3.	https://osf.io/eam3y/

The pieces functions are:

• The pieces (3) FiberHolder serves to attach one end of each of the fibers.
• The pieces (2) UnionPiece join each of the fiber holders using M3 bolts and nuts. This part works to give separation and fix the angle between each of the fibers.
• BasePiece (piece number 4) supports the end of one fiber per arm and joins the system structure to the Base (5).
• Base (5) is the base platform for fixing all the components.
• The FiberMatrix (6) arranges the opposite end of all the optical fibers and fixes them to the Base (5).
• Finally, the centerpiece CenterPiece (1) holds one optical fiber in the overhead direction and joins the eight arms using M3 bolts and nuts.

The previous setup brings a total of 33 optical fibers, 24 UnionPiece, 24 PieceHolder, 8 BasePiece, one Base, and one acrylic semispherical cover to protect the system.

## 5 Bill of materials

This section presents a list of the parts that must be purchased for the manufacture of this device, including the current cost and a possible supplier. It is important to note that the acquisition of the images can be performed in many ways by the users of this system; in the case of the proposed validation, this system uses a Raspberry Pi only as an example. Table 2 shows a summary of the materials required for the implementation of this device.

**Table 2:** Bill of materials

Designator	Component	Qty	Unit cost	Total cost	Source of material
PLA filament	Structural	0.5	$22.99	$11.50	t.ly/CDZ2
Acrilyc sheet	Structural	1	$9.99	$9.99	t.ly/WpUL
Acrilyc dome 10”	Protection	1	$39.99	$39.99	t.ly/9ii2
Solid core fiber optic	Sensor	0.2	$42.69	$8.54	t.ly/YCt3
Raspberry Pi 3 Kit	Processing	1	$98.95	$98.95	t.ly/o7aB
Raspberry Pi Camera	Processing	1	$34.95	$34.95	t.ly/ie2G
			Total	$203.91	

## 6 Build instructions

**Fig. 5:**
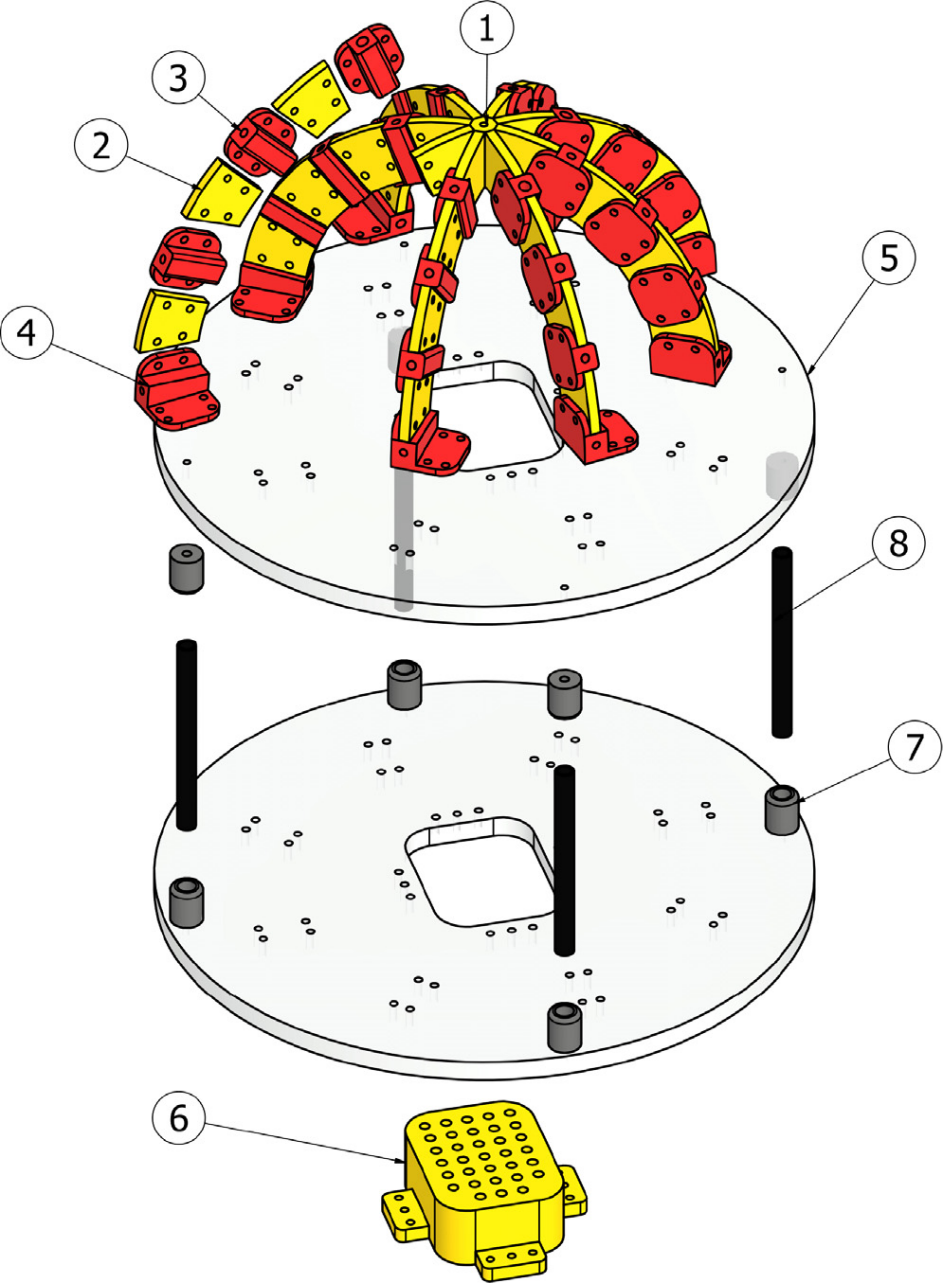
Total assembly of the climate station.

First, the base device must be cut (acrylic base), this corresponds to a 30 cm diameter acrylic circle; two of these pieces will be used. Then, the following pieces must be 3D printed: *CenterPiece*, *UnionPiece*, *FiberHolder*, *BasePiece*, *FiberMatrix*, and *BarEnd*. For that purpose, either PLA or Acrylonitrile butadiene-styrene (ABS) can be used. In this case, the parts were printed using PLA. For the assembly, the first step is to join the semi-arches formed by three union pieces (UnionPiece), three fiber holders (FiberHolder), and one arch base (BasePiece). The device is formed by eight semi-arches that must be attached to the central piece (CenterPiece); thus this structure has the shape of a dome with a diameter of 23 cm. Next, the arches assembly is positioned above the acrylic base, and the fiber matrix (FiberMatrix) is attached to the free base. Those two main parts are then attached using 10 cm aluminum tubes with 8 mm diameter, these tubes have cylindrical pieces printed in 3D (Barnt) at the ends to fix them to the acrylic bases. Finally, for all the joints M3 screws are used. Table 3 describes each of the elements in the mechanical assembly, and Fig. 5 provides a visual description of the assembly.

**Table 3:** Required of elements for the main assembly

Part number	Part Name	Quantity
1	CencerPiece	1
2	UnionPiece	24
3	FiberHolder	24
4	BasePiece	8
5	acrylic base	2
6	FiberMatrix	1
7	BarEnd	8
8	aluminium tube	4

Once the assembly is finished, 33 optical fibers of 23 cm long are cut, the end of the fibers is inserted through the holes in the centerpiece (CenterPiece), the fiber holders (FiberHolder) and the bases of the arch (BasePiece). This forces the fibers to be distributed every $22.5^{o}$ in the eight semi-arcs to form four arcs of $360^{o}$. Finally, the other end of the fibers is inserted into the matrix, where the camera will be located for taking the light intensities.

## 7 Operation instructions

Taking into account that this device is an optomechanical system, its operation has no complexity. After being assembled, it is only necessary to perform the device installation in the desired site. It is recommended to install the device in a flat surface, which avoids the need for inclination corrections.

The geographical coordinates and orientation can be obtained using an external equipment (a smartphone for example), since both orientation and location should not be changed during a round of data acquisition. Avoiding sensors for those measurements was a design decision to reduce the cost of the final prototype. Finally, after the device is located, the data acquisition can be accomplished.

## 8 Validation and characterization

**Fig. 6:**
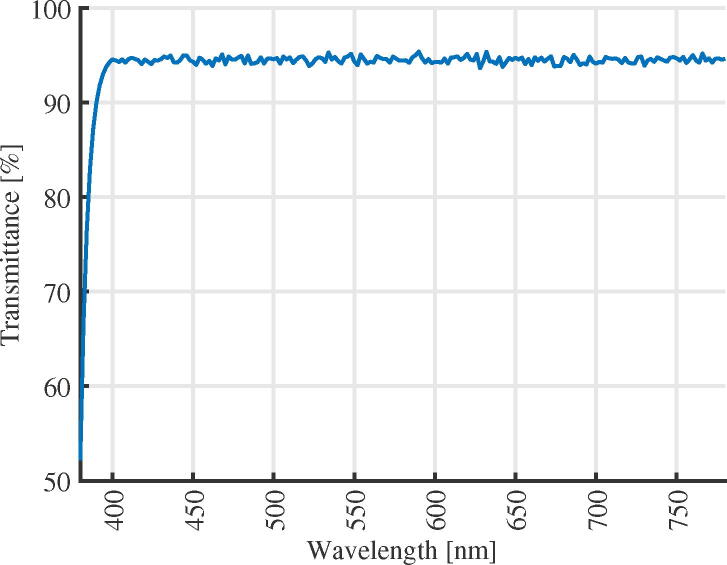
Acrylic transmittance.

The first validation is focused on the optical characteristics of the acrylic dome, which is needed to ensure that the dome will not affect significantly the irradiance measurements. This type of validation has been performed in other works for the polymethyl methacrylate material using spectrometers [21]. This work follows the same principle, using the handheld spectrometer OHSP350 to perform measurements in the field. The dome transmittance was calculated by taking spectral measurements in both outside and inside of the dome, which produce the transmittance curve reported in Fig. 6. Those results report that the dome transmittance is close to 95% in the 400–780 nm region, with a transmittance decay in the range 380–400 nm however, since the device is intended for power measurement, such a decay does not introduce a significantly error.

**Fig. 7:**
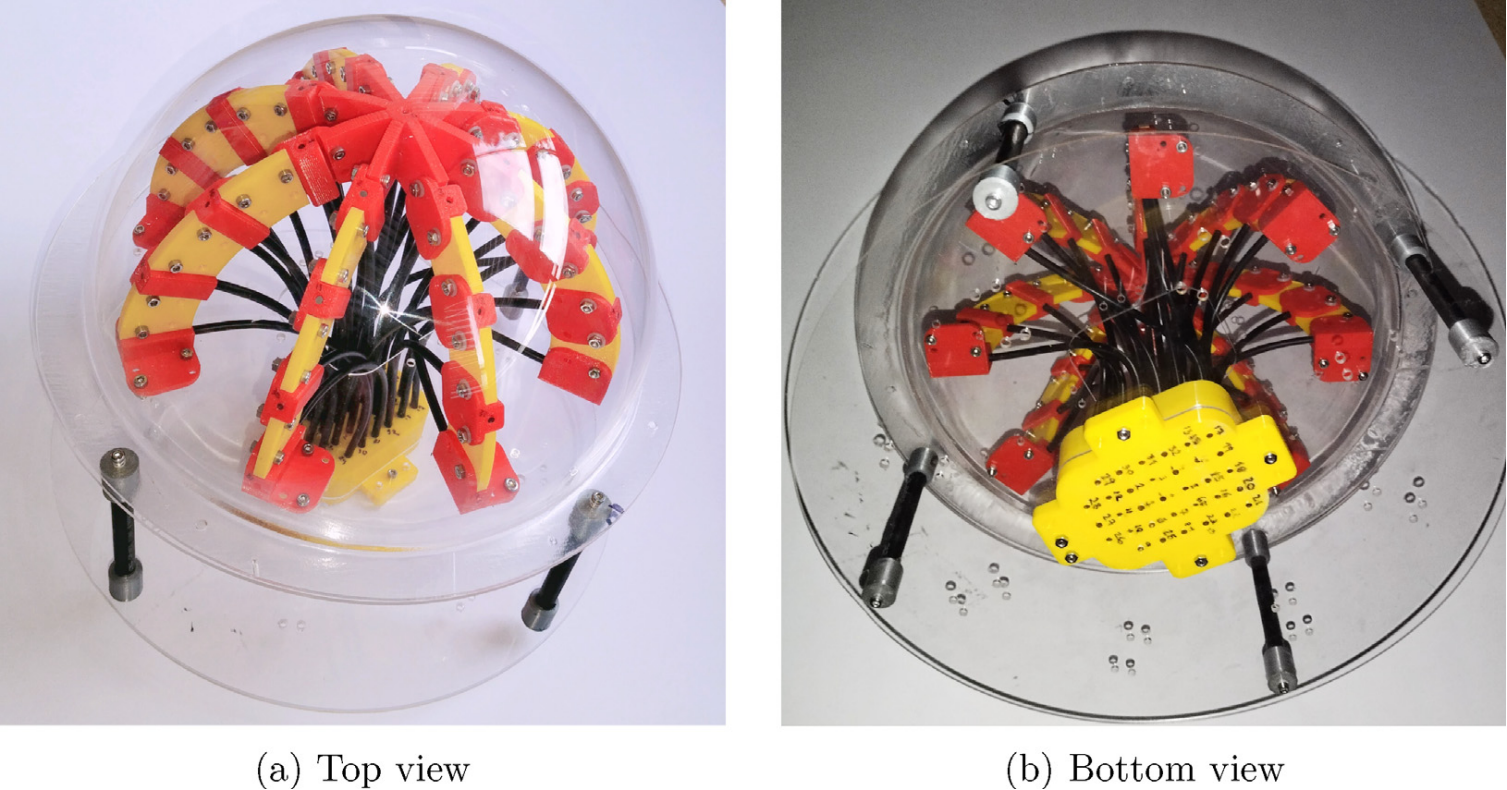
Photographs of the assembled system.

Fig. 7 shows photographs of the assembled system. In Fig. 7a a top view is shown, where the acrylic dome that protects the assembly of the elements is identified. Fig. 7b shows an interior view of the device, where the matrix collecting the optical fibers is observed. In the photographs it also observed the location of the optical fibers, which are covered with PVC for protection and to avoid interference from adjacent fibers. In addition the tips of the fibers collecting light are aligned by the pieces in the arches, and the other ends arrive at the yellow matrix at the base.

**Fig. 8:**
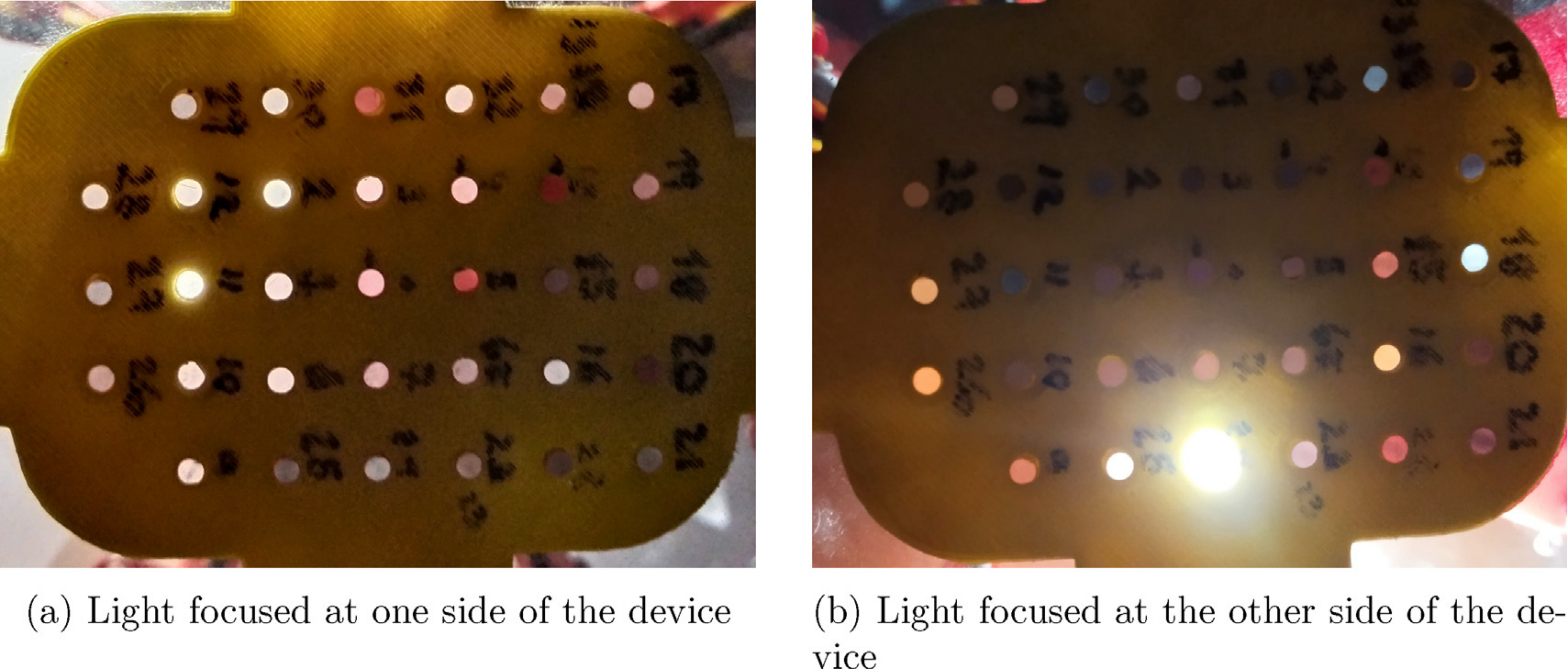
Example images of the optical fiber matrix.

Fig. 8a and b show two example images of the optical fiber matrix with different sunlight direction and intensity. Those figures show the light transmission capacity of the optical fibers, which enables to detect multiple sources (fibers) with a single RGB camera. In those figures some positions show red light, this is because the light is passing laterally through the PLA that supports the fiber. Such an effect is not detrimental for the measurements since the illumination intensity is normalized.

Then, the images captured by the camera are processed by a raspberry microprocessor. For that purpose, a data mask was built around the position of each fiber into the image, which isolates each particular fiber in a region of 30x30 pixels. Therefore, 33 data mask were stored in the raspberry, which are used to extract the region of the image around each fiber, and such an information is stored in a table and correlated with the ID of the fiber. Then, the average intensity level of each 30x30 pixel region is evaluated for each measurement, i.e. for each captured image. This is a simple but effective process as it will be demonstrated in the experimental validation.

**Fig. 9:**
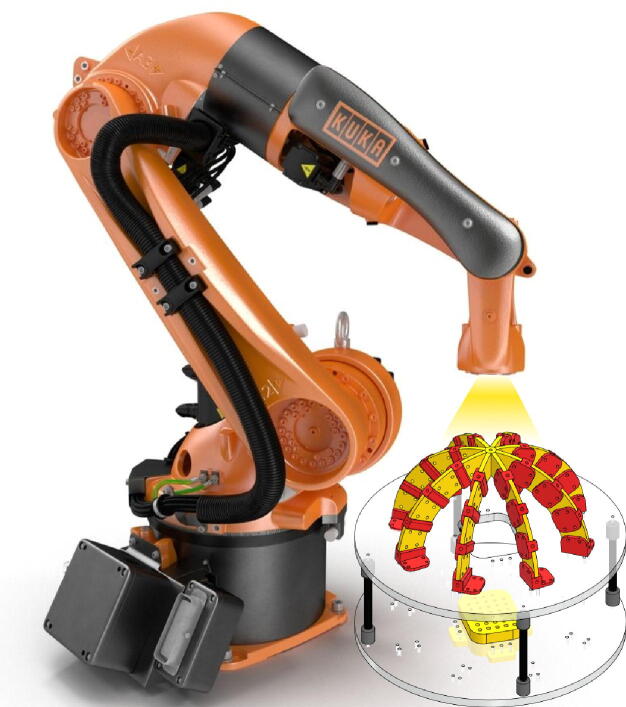
Testing scheme based on a position-controlled illumination system (KUKA robot and Halogen Light) used to evaluate the response of each optical fiber.

**Fig. 10:**
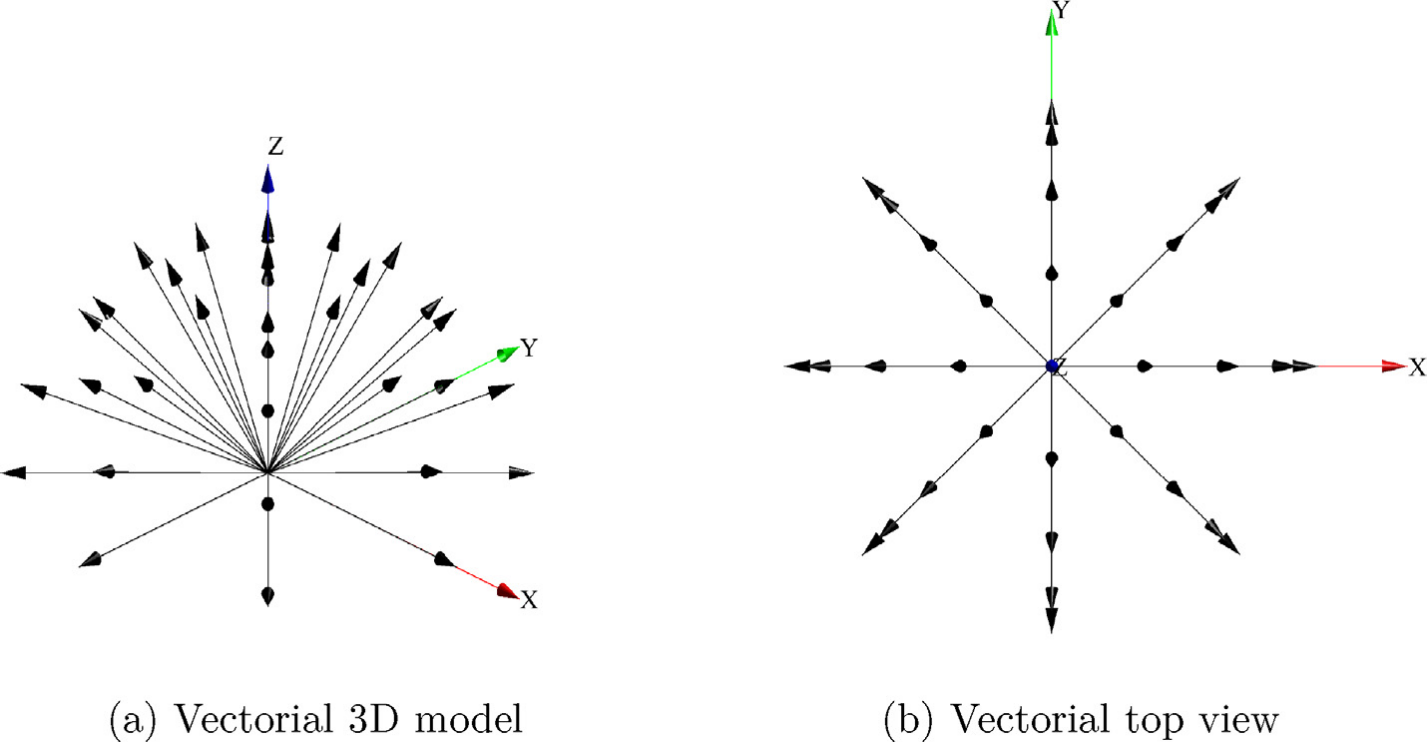
Vectorial representation of the optical fibers.

To validate the operation of the system, a halogen directional light source attached to a KUKA ARC5 robotic manipulator was used, as depicted in Fig. 9. This test system performs repeatable positions for the light source with a precision of 0.04 mm, thus allowing a direct illumination of each optical fiber in the matrix. Fig. 10a and b show the 33 vectors representing the direction of each of the optical fiber in the proposed system.

**Fig. 11:**
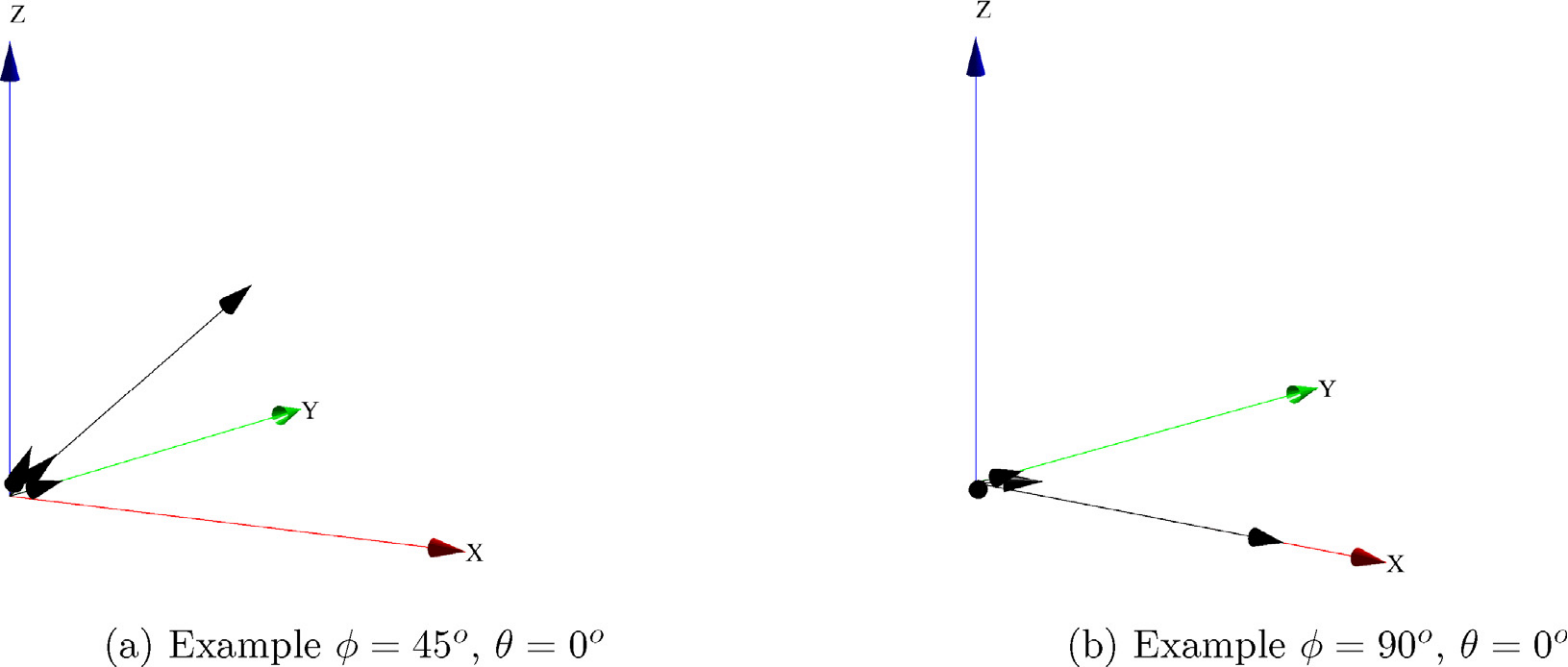
System response to different positions of the light source.

Since each optical fiber gives the intensity of particular vector, as observed in Fig. 10, the vectorial sum of those intensities allows the calculation of the light source position. This is illustrated in Fig. 11a, which shows the system response when the light source is located by the robot at the coordinates $\phi =45^{o},\theta =0^{o}$. Similarly, Fig. 11b shows the system response when the light source is located at the coordinates $\phi =90^{o},\theta =0^{o}$.

Finally, Table 4 shows the results of the validation exercise. Since the validation consisted on directly illuminating each optical fiber (the indexes were previously defined in Fig. 1b), each column of the table reports the the normalized value of the intensity response of each of the 33 fibers. For example, the first column (index  = 0) corresponds to the light source located at the first fiber, where fiber 0 has a normalized illumination of 0.9, while the adjacent fibers (1 to 8) report a much lower illumination, and the other fibers (9 to 32) do not report any illumination. The experiments on the other 32 positions report similar results, where the adjacent fibers only shows between 1 % and 5 % of the normalized illumination reported by the fiber under direct test. This small error is caused, in part, by the cone shape of the light provided by the halogen source. In any case, since similar errors are present in all directions around the fiber under test, and those errors are very small, the detection of the light orientation is not significantly affected.

**Table 4:** Validation of the device

Index	0	1	2	3	4	5	6	7	8	9	10	11	12	13	14	15	16	17	18	19	20	21	22	23	24	25	26	27	28	29	30	31	32
0	0.90	0.05	0.04	0.05	0.04	0.05	0.04	0.03	0.06	0.00	0.00	0.00	0.00	0.00	0.00	0.00	0.00	0.00	0.00	0.00	0.00	0.00	0.00	0.00	0.00	0.00	0.00	0.00	0.00	0.00	0.00	0.00	0.00
1	0.05	0.92	0.03	0.00	0.00	0.00	0.00	0.00	0.05	0.00	0.04	0.00	0.00	0.00	0.00	0.00	0.00	0.00	0.00	0.00	0.00	0.00	0.00	0.00	0.00	0.00	0.00	0.00	0.00	0.00	0.00	0.00	0.00
2	0.04	0.03	0.93	0.05	0.00	0.00	0.00	0.00	0.00	0.00	0.00	0.05	0.00	0.00	0.00	0.00	0.00	0.00	0.00	0.00	0.00	0.00	0.00	0.00	0.00	0.00	0.00	0.00	0.00	0.00	0.00	0.00	0.00
3	0.04	0.00	0.04	0.96	0.04	0.00	0.00	0.00	0.00	0.00	0.00	0.00	0.04	0.00	0.00	0.00	0.00	0.00	0.00	0.00	0.00	0.00	0.00	0.00	0.00	0.00	0.00	0.00	0.00	0.00	0.00	0.00	0.00
4	0.03	0.00	0.00	0.05	0.97	0.05	0.00	0.00	0.00	0.00	0.00	0.00	0.00	0.05	0.00	0.00	0.00	0.00	0.00	0.00	0.00	0.00	0.00	0.00	0.00	0.00	0.00	0.00	0.00	0.00	0.00	0.00	0.00
5	0.04	0.00	0.00	0.00	0.50	0.94	0.05	0.00	0.00	0.00	0.00	0.00	0.00	0.00	0.06	0.00	0.00	0.00	0.00	0.00	0.00	0.00	0.00	0.00	0.00	0.00	0.00	0.00	0.00	0.00	0.00	0.00	0.00
6	0.05	0.00	0.00	0.00	0.00	0.05	0.96	0.07	0.00	0.00	0.00	0.00	0.00	0.00	0.00	0.07	0.00	0.00	0.00	0.00	0.00	0.00	0.00	0.00	0.00	0.00	0.00	0.00	0.00	0.00	0.00	0.00	0.00
7	0.02	0.00	0.00	0.00	0.00	0.00	0.05	0.95	0.05	0.00	0.00	0.00	0.00	0.00	0.00	0.00	0.06	0.00	0.00	0.00	0.00	0.00	0.00	0.00	0.00	0.00	0.00	0.00	0.00	0.00	0.00	0.00	0.00
8	0.05	0.05	0.00	0.00	0.00	0.00	0.00	0.05	0.97	0.03	0.00	0.00	0.00	0.00	0.00	0.00	0.00	0.00	0.00	0.00	0.00	0.00	0.00	0.00	0.00	0.00	0.00	0.00	0.00	0.00	0.00	0.00	0.00
9	0.00	0.00	0.00	0.00	0.00	0.00	0.00	0.00	0.05	0.95	0.06	0.00	0.00	0.00	0.00	0.00	0.05	0.00	0.00	0.00	0.00	0.00	0.00	0.00	0.00	0.00	0.03	0.00	0.00	0.00	0.00	0.00	0.00
10	0.00	0.04	0.00	0.00	0.00	0.00	0.00	0.00	0.00	0.05	0.96	0.04	0.00	0.00	0.00	0.00	0.00	0.00	0.00	0.00	0.00	0.00	0.00	0.00	0.00	0.00	0.00	0.00	0.04	0.00	0.00	0.00	0.00
11	0.00	0.00	0.04	0.00	0.00	0.00	0.00	0.00	0.00	0.00	0.05	0.94	0.05	0.00	0.00	0.00	0.00	0.00	0.00	0.00	0.00	0.00	0.00	0.00	0.00	0.00	0.00	0.00	0.00	0.00	0.04	0.00	0.00
12	0.00	0.00	0.00	0.06	0.00	0.00	0.00	0.00	0.00	0.00	0.00	0.05	0.97	0.06	0.00	0.00	0.00	0.00	0.00	0.00	0.00	0.00	0.00	0.00	0.00	0.00	0.00	0.00	0.00	0.00	0.00	0.00	0.03
13	0.00	0.00	0.00	0.00	0.05	0.00	0.00	0.00	0.00	0.00	0.00	0.00	0.05	0.94	0.05	0.00	0.00	0.00	0.00	0.04	0.00	0.00	0.00	0.00	0.00	0.00	0.00	0.00	0.00	0.00	0.00	0.00	0.00
14	0.00	0.00	0.00	0.00	0.00	0.07	0.00	0.00	0.00	0.00	0.00	0.00	0.00	0.05	0.95	0.05	0.00	0.00	0.00	0.00	0.03	0.00	0.00	0.00	0.00	0.00	0.00	0.00	0.00	0.00	0.00	0.00	0.00
15	0.00	0.00	0.00	0.00	0.00	0.00	0.05	0.00	0.00	0.00	0.00	0.00	0.00	0.00	0.05	0.96	0.05	0.00	0.00	0.00	0.00	0.00	0.04	0.00	0.00	0.00	0.00	0.00	0.00	0.00	0.00	0.00	0.00
16	0.00	0.00	0.00	0.00	0.00	0.00	0.00	0.05	0.00	0.06	0.00	0.00	0.00	0.00	0.00	0.05	0.95	0.00	0.00	0.00	0.00	0.00	0.00	0.00	0.04	0.00	0.00	0.00	0.00	0.00	0.00	0.00	0.00
17	0.00	0.00	0.00	0.00	0.00	0.00	0.00	0.00	0.00	0.00	0.00	0.00	0.00	0.00	0.00	0.00	0.00	0.94	0.01	0.00	0.00	0.00	0.00	0.00	0.00	0.00	0.00	0.00	0.00	0.00	0.00	0.01	0.04
18	0.00	0.00	0.00	0.00	0.00	0.00	0.00	0.00	0.00	0.00	0.00	0.00	0.00	0.00	0.00	0.00	0.00	0.02	0.95	0.05	0.00	0.00	0.00	0.00	0.00	0.00	0.00	0.00	0.00	0.00	0.00	0.00	0.00
19	0.00	0.00	0.00	0.00	0.00	0.00	0.00	0.00	0.00	0.00	0.00	0.00	0.00	0.05	0.00	0.00	0.00	0.00	0.03	0.97	0.01	0.02	0.00	0.00	0.00	0.00	0.00	0.00	0.00	0.00	0.00	0.00	0.01
20	0.00	0.00	0.00	0.00	0.00	0.00	0.00	0.00	0.00	0.00	0.00	0.00	0.00	0.00	0.05	0.00	0.00	0.00	0.00	0.02	0.96	0.05	0.01	0.00	0.00	0.00	0.00	0.00	0.00	0.00	0.00	0.00	0.00
21	0.00	0.00	0.00	0.00	0.00	0.00	0.00	0.00	0.00	0.00	0.00	0.00	0.00	0.00	0.00	0.00	0.00	0.00	0.02	0.00	0.05	0.90	0.00	0.01	0.00	0.00	0.00	0.00	0.00	0.00	0.00	0.00	0.00
22	0.00	0.00	0.00	0.00	0.00	0.00	0.00	0.00	0.00	0.00	0.00	0.00	0.00	0.00	0.00	0.05	0.00	0.00	0.00	0.00	0.01	0.01	0.91	0.04	0.01	0.00	0.00	0.00	0.00	0.00	0.00	0.00	0.00
23	0.00	0.00	0.00	0.00	0.00	0.00	0.00	0.00	0.00	0.00	0.00	0.00	0.00	0.00	0.00	0.00	0.00	0.00	0.00	0.00	0.00	0.00	0.05	0.94	0.00	0.01	0.00	0.00	0.00	0.00	0.00	0.00	0.00
24	0.00	0.00	0.00	0.00	0.00	0.00	0.00	0.00	0.00	0.00	0.00	0.00	0.00	0.00	0.00	0.00	0.06	0.00	0.00	0.00	0.00	0.00	0.01	0.00	0.96	0.04	0.01	0.00	0.00	0.00	0.00	0.00	0.00
25	0.00	0.00	0.00	0.00	0.00	0.00	0.00	0.00	0.00	0.00	0.00	0.00	0.00	0.00	0.00	0.00	0.00	0.00	0.00	0.00	0.00	0.00	0.00	0.01	0.05	0.96	0.00	0.01	0.00	0.00	0.00	0.00	0.00
26	0.00	0.00	0.00	0.00	0.00	0.00	0.00	0.00	0.00	0.04	0.00	0.00	0.00	0.00	0.00	0.00	0.00	0.00	0.00	0.00	0.00	0.00	0.00	0.00	0.01	0.00	0.95	0.03	0.01	0.00	0.00	0.00	0.00
27	0.00	0.00	0.00	0.00	0.00	0.00	0.00	0.00	0.00	0.00	0.00	0.00	0.00	0.00	0.00	0.00	0.00	0.00	0.00	0.00	0.00	0.00	0.00	0.00	0.00	0.02	0.04	0.94	0.00	0.01	0.00	0.00	0.00
28	0.00	0.00	0.00	0.00	0.00	0.00	0.00	0.00	0.00	0.00	0.04	0.00	0.00	0.00	0.00	0.00	0.00	0.00	0.00	0.00	0.00	0.00	0.00	0.00	0.00	0.00	0.01	0.00	0.97	0.03	0.02	0.00	0.00
29	0.00	0.00	0.00	0.00	0.00	0.00	0.00	0.00	0.00	0.00	0.00	0.00	0.00	0.00	0.00	0.00	0.00	0.00	0.00	0.00	0.00	0.00	0.00	0.00	0.00	0.00	0.00	0.01	0.04	0.96	0.00	0.02	0.00
30	0.00	0.00	0.00	0.00	0.00	0.00	0.00	0.00	0.00	0.00	0.00	0.05	0.00	0.00	0.00	0.00	0.00	0.00	0.00	0.00	0.00	0.00	0.00	0.00	0.00	0.00	0.00	0.00	0.01	0.00	0.95	0.04	0.01
31	0.00	0.00	0.00	0.00	0.00	0.00	0.00	0.00	0.00	0.00	0.00	0.00	0.00	0.00	0.00	0.00	0.00	0.02	0.00	0.00	0.00	0.00	0.00	0.00	0.00	0.00	0.00	0.00	0.00	0.01	0.04	0.94	0.00
32	0.00	0.00	0.00	0.00	0.00	0.00	0.00	0.00	0.00	0.00	0.00	0.00	0.05	0.00	0.00	0.00	0.00	0.01	0.00	0.01	0.00	0.00	0.00	0.00	0.00	0.00	0.00	0.00	0.00	0.00	0.01	0.00	0.94

The main features and limitations of the proposed device are:

• The proposed system is a low cost device, simplifies the electronic part of the implementation and allows a relatively high measurement resolution.
• Due to the numerical aperture of the optical fiber, the measurement has an error due to light coming from different angles.
• The proposed system can be easily scaled up by adjusting the design, with a very low increment in cost due to the additional printed parts and fiber optics.

## Ethics statements

No human or animal studies were conducted in this work.

## Declaration of Competing Interest

The authors declare that they have no known competing financial interests or personal relationships that could have appeared to influence the work reported in this paper.
